# Structural analysis of the Aβ(11–42) amyloid fibril based on hydrophobicity distribution

**DOI:** 10.1007/s10822-019-00209-9

**Published:** 2019-07-10

**Authors:** Irena Roterman, Dawid Dułak, Małgorzata Gadzała, Mateusz Banach, Leszek Konieczny

**Affiliations:** 1 Department of Bioinformatics and Telemedicine, Jagiellonian University – Medical College, Łazarza 16, 31-530 Kraków, Poland; 2 ABB Business Services Sp. z o.o., Żegańska 1, 04-713 Warsaw, Poland; 3 ACK – Cyfronet AGH, Nawojki 11, 30-150 Kraków, Poland; 4 Present Address: Schibsted Tech Polska Sp. z o. o., Armii Krajowej 28, 30-150 Kraków, Poland; 5 Chair of Medical Biochemistry, Jagiellonian University – Medical College, Kopernika 7, 31-034 Kraków, Poland

**Keywords:** Amyloidosis, Fibrillation, Aβ(11–42), Aβ(1–42)

## Abstract

The structure of the Aβ(11–42) amyloid available in PDB makes possible the molecular analysis of the specificity of amyloid formation. This molecule (PDB ID 2MVX) is the object of analysis. This work presents the outcome of in silico experiments involving various alternative conformations of the Aβ(11–42) sequence, providing clues as to the amylodogenecity of its constituent fragments. The reference structure (PDB) has been compared with folds generated using I-Tasser and Robetta—the strongest contenders in the CASP challenge. Additionally, a polypeptide which matches the Aβ(11–42) sequence has been subjected to folding simulations based on the fuzzy oil drop model, which favors the production of a monocentric hydrophobic core. Computer simulations yielded 15 distinct structural forma (five per software package), which, when compared to the experimentally determined structure, allow us to study the role of structural elements which cause an otherwise globular protein to transform into an amyloid. The unusual positions of hydrophilic residues disrupting the expected hydrophobic core and propagating linearly along the long axis of fibril is recognized as the seed for amyloidogenic transformation in this polypeptide. This paper discusses the structure of the Aβ(11–42) amyloid fibril, listed in PDB under ID 2MXU (fragment od Aβ(1–42) amyloid).

## Introduction

The volume of published papers which focus on amyloids is rapidly growing. Literature reviews [1] provide an up-to-date overview of current trends in protein misfolding research. This broad field encompasses various specific issues, such as the genetic underpinnings of amyloidogenesis and molecular studies [2], including protein folding simulations. Analysis of amyloid structures has to deal with the dynamics and flexibility of proteins, which must be capable of specific interactions with their intended ligands and substrates [3, 4]. The emergence of amyloids is linked to the peculiarities of the folding process, which still awaits a comprehensive theoretical description—despite many decades of research [5].

In order to study such phenomena, we require techniques which would enable us to track the intermediate phases of folding. While traditional NMR is a useful tool, it requires soluble molecules [6], and that presents a problem when studying the pathogenicity of amyloids [7–9]. Much progress has recently been made owing to introduction of solid-state NMR [10]. One example is the elucidation of the structure of Aβ(1–42), and particularly of its Aβ(11–42) fragment, which is now listed in PDB [11]. This amyloid is the focus of the presented work. We subjected it to analysis from the point of view of hydrophobicity distribution. The presented work follows upon the results presented in [12], where we single out fragments exhibiting specific deviations from the theoretical (“idealized”) distribution of hydrophobicity expected in a globular protein and mathematically defined by a 3D Gaussian [13–15].

The Gaussian distribution peaks at the center of an ellipsoid capsule. Its values decrease along with distance from the center, reaching nearly 0 on the surface. When the size of the capsule is adjusted to encapsulate the molecule in question, the corresponding Gaussian yields the theoretical (expected) values of hydrophobicity at any point within the protein body. This theoretical distribution is subsequently compared with the observed distribution of hydrophobicity, which depends on inter-residual interactions (themselves dependent on the mutual separation and intrinsic hydrophobicity of interacting residues). Differences between both distributions manifest themselves as either excess hydrophobicity or hydrophobicity deficiencies in specific areas of the protein body. The former—if present on the surface—mark protein complexation interfaces [16], while the latter are typically associated with ligand binding cavities [17] which enable the protein to perform its biological function [18].

To-date studies of amyloids point to a specific disagreement between the observed distribution of hydrophobicity and the theoretical (monocentric) Gaussian. In place of a central core, we are faced with linear propagation of repetitive patterns stretching along the fibril’s main axis. Several fragments of the Aβ(1–42) polypeptide have been identified as amyloid seeds—this includes the fragments at 11–16, 16–22 and 22–28 [12]. Consequently, our analysis will focus on these fragments in particular.

The Aβ(11–42) sequence has been used as input for protein folding simulation toolkits, including I-Tasser [19–22] and Robetta [23, 24]—both highly ranked in the CASP competition [25, 26]. Structures produced by both packages were assessed from the point of view of hydrophobicity distribution. In this respect, they proved to be highly diverse, ranging from near-globular to amyloid-like. In addition, we also generated several reference structures using software based on the fuzzy oil drop model (FOD), augmenting optimization of nonbonding interactions with alignment with an external (Gaussian) hydrophobic force field.

Each of the presented toolkits produced five structures, enabling us to compile a ranking list sorted by increasing differences between the observed structure and the theoretical Gaussian. This, in turn, shows how progressive deviations from the monocentric hydrophobic core model eventually cause the polypeptide chain to transform into a fibril, marked by alternating bands of high and low hydrophobicity. This analysis can be related to the reported polymorphism of amyloid structures [27].

This is why this paper can be treated as in silico experiment.

## Materials and methods

### Protein under consideration

Our analysis focused on the Aβ(11–42) amyloid listed in PDB under ID 2MXU [11]. It represents the Aβ(1–42) protein devoid of its N-terminal fragment. The PDB structure comprises 12 separate polypeptides arranged into a fibril. This structural form is treated as reference one for all models delivered by mentioned programs. The status of complete fibrils, status of the chain as part of the fibril and one selected chain treated as individual structural unit are taken as the reference objects for those generated in silico.

### Folding of Aβ(11–42)

The Aβ(11–42) polypeptide was subjected to in silico folding simulations using two state-of-the-art protein folding toolkits: I-Tasser [19–22] and Robetta [23, 24]. Both packages consistently obtain high marks in the CASP [25] challenge and have been singled out as the most reliable computational tools currently available for this purpose [22, 23]. The diversity of the resulting structures has its roots in the reported polymorphism of amyloid forms [27].

In addition to the above, our study set was extended with five structures generated by a custom toolkit based on the fuzzy oil drop model, which acknowledges the influence of the aqueous solvent upon the chain in question. This influence is modeled as an external force field with a Gaussian distribution, promoting internalization of hydrophobic residues along with exposure of hydrophilic residues on the surface [13–15]. As the application requires a starting structure (referred to as the Early Stage intermediate) we used the 2MXU structure as its input.

All resulting folds (15 in total; five per application—pursuant to CASP criteria), along with the structure available in PDB, were subject to analysis of the shape of their hydrophobic cores in two separate contexts: as a composite fibril and as an individual chain from the 2MXU file.

FOD model was applied for protein folding for selected targets available in CASP6 (2004) [24]. We submitted models for 23 targets with the highest DTT_TS value equal to 41.98.

### Fuzzy oil drop model (FOD)

The status of the hydrophobic core is expressed using the RD value which bases on the fuzzy oil drop model. This coefficient is calculated separately for two distinct variants: RD(T-O-R), which compares the observed distribution (O) with two reference distributions: theoretical (T) and uniform (R), and RD(T-O-H), where the uniform distribution is replaced by a distribution which reflects the distribution expressed by intrinsic hydrophobicity of each residue. The definition of these parameters will be given below in the next part of Materials and Methods. In addition, three correlation coefficients of the above hydrophobicity distributions are computed: H versus T, T versus O and H versus O called as HvT, TvO and HvO in this paper.

Since a detailed presentation of the fuzzy oil drop model can be found in [13–15] we will limit ourselves to a brief recapitulation of its core concepts.

The input molecule is encapsulated in an ellipsoid used to calibrate the 3D Gaussian function, which, in turn, yields theoretical values of hydrophobicity at arbitrary points in the protein body. In contrast, the observed distribution depends on inter-residual interactions (as described in [28]). Both distributions (T and O) are measured at specific points which correspond to the so-called effective atoms (averaged-out positions of all atoms comprising each residue). In addition, each residue is assumed to represent certain intrinsic hydrophobicity as listed in [19], which is also used in observed hydrophobicity calculations.

In order to meaningfully compare alternative distributions, we apply the so-called Kullback–Leibler divergence entropy formula [29]. Since the result produced by this formula is a measure of entropy, it cannot be interpreted on its own—instead, it requires a reference value. This is why, in addition to T, we introduce another reference distribution denoted R, which stands from random. It is a uniform distribution which assigns every residue under consideration a hydrophobicity of 1/N where is N the number of these residues. Under these assumptions the RD value expresses the “closeness” of O to either T or R. Since—as discussed in [12]—in amyloid structures the observed distribution is dominated by the intrinsic properties of each participating residue, we also define another type of reference, denoted H, which reflects the intrinsic hydrophobicity of each amino acid in the input chain. This results in two distinct values of RD: one for the T-O-R variant and one for the T-O-H variant. In the first case, a RD value greater or equal than 0.5 is taken as indication that protein’s observed hydrophobicity profile does not follow the 3D Gaussian distribution, while in the other—that it explicitly follows the intrinsic model (H).

As already mentioned, our comparative analysis also relies on three distinct correlation coefficients, providing a pairwise comparison of all distributions: HvT, TvO and HvO (v stands from “versus”). Together, these coefficients express the influence of intrinsic hydrophobicity upon the structure of the hydrophobic core.

Globular proteins are usually closely aligned with T (this is particularly true for domains [30]), which means that hydrophobic residues are internalized while hydrophilic residues appear on the surface. Such conditions emerge as a result of “cooperation” between residues in an attempt to produce a common core. On the other hand, when residues act in a “selfish” manner, without cooperative tendencies, the result is a high value of HvO coupled with low (or even negative) values of both TvO and HvT. This suggests that the structure does not contain a monocentric core, and instead may exhibit other—in case of amyloid: linear propagation of repetitive patterns of hydrophobicity. Consequently, analysis of RD (together with the aforementioned correlation coefficients) may reveal progressive dilution of the hydrophobic core in favor of an entirely different structural pattern.

In order to further identify strongly amyloidogenic fragments, the above coefficients were also calculated for specific fragments of the input polypeptide.

Folding simulations were performed using computational resources provided by the Cyfronet AGH—Academic Computing Center within the PL-Grid infrastructure. In fuzzy oil drop simulations the optimization of nonbonding interactions was carried out using the GROMACS package (also provided by Cyfronet) [31, 32].

## Results

### Structure of the Aβ(11–42) fibril

**Fig. 1:**
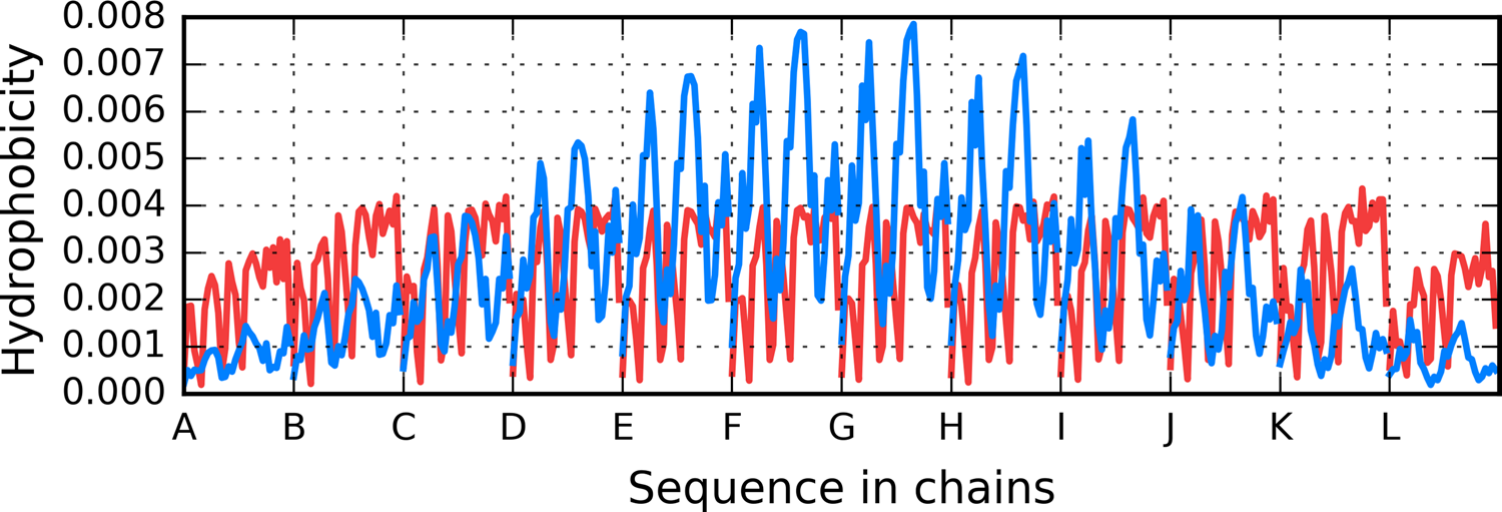
Theoretical (T: blue) and observed (O: red) hydrophobicity distributions in the Aβ(11–42) fibril

The structure of the Aβ(11–42) fibril may be assessed on the basis of T and O hydrophobicity distributions, as illustrated in Fig. 1. The figure reveals typical discordance between both distributions, with the observed hydrophobicity remaining high on the surface of the complex (contrary to expectations). In addition, a characteristic sinusoidal pattern is observed—the hallmark of a complex comprising multiple identical subunits arranged in a linear fashion, with alternating bands of high and low hydrophobicity exposed on the surface.

RD and correlation coefficients characterizing the fibrillary form will be discussed further on, in conjunction with the analysis of structures generated by in silico folding models.

**Fig. 2:**
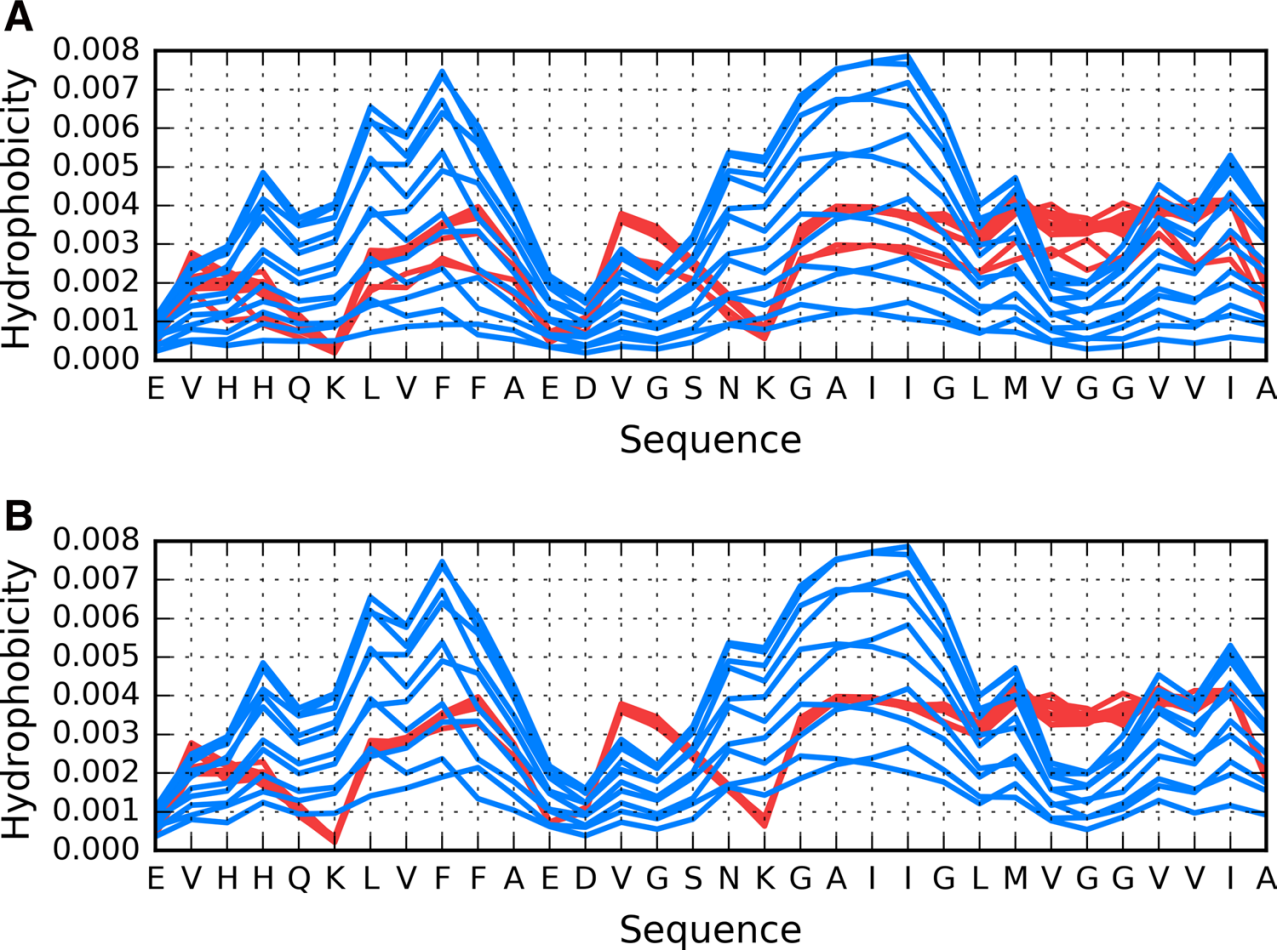
Theoretical (T: blue) and observed (O: red) hydrophobicity distributions in the Aβ(11–42) fibril presented in an overlapped mode (with all chains sharing the X axis): **A** all chains present in the fibril; **B** without one outlying chain from each end of the structure

The distribution of T shown in Fig. 1 reveals the characteristic concentration of hydrophobicity in the central part of fibril which is not followed by O distribution which represents sinusoid-like distribution along the whole fibril. The red lines do not represent the different O distributions. Only two O profiles can bees in Fig. 1a. This is due to overlapping of almost identical profiles for central polypeptides. Elimination of border polypeptide chains (chains A and L) visualizes it very clear (Fig. 2b). The T distributions for these polypeptides still represent different form depending of the position of polypeptide chain under consideration.

**Fig. 3:**
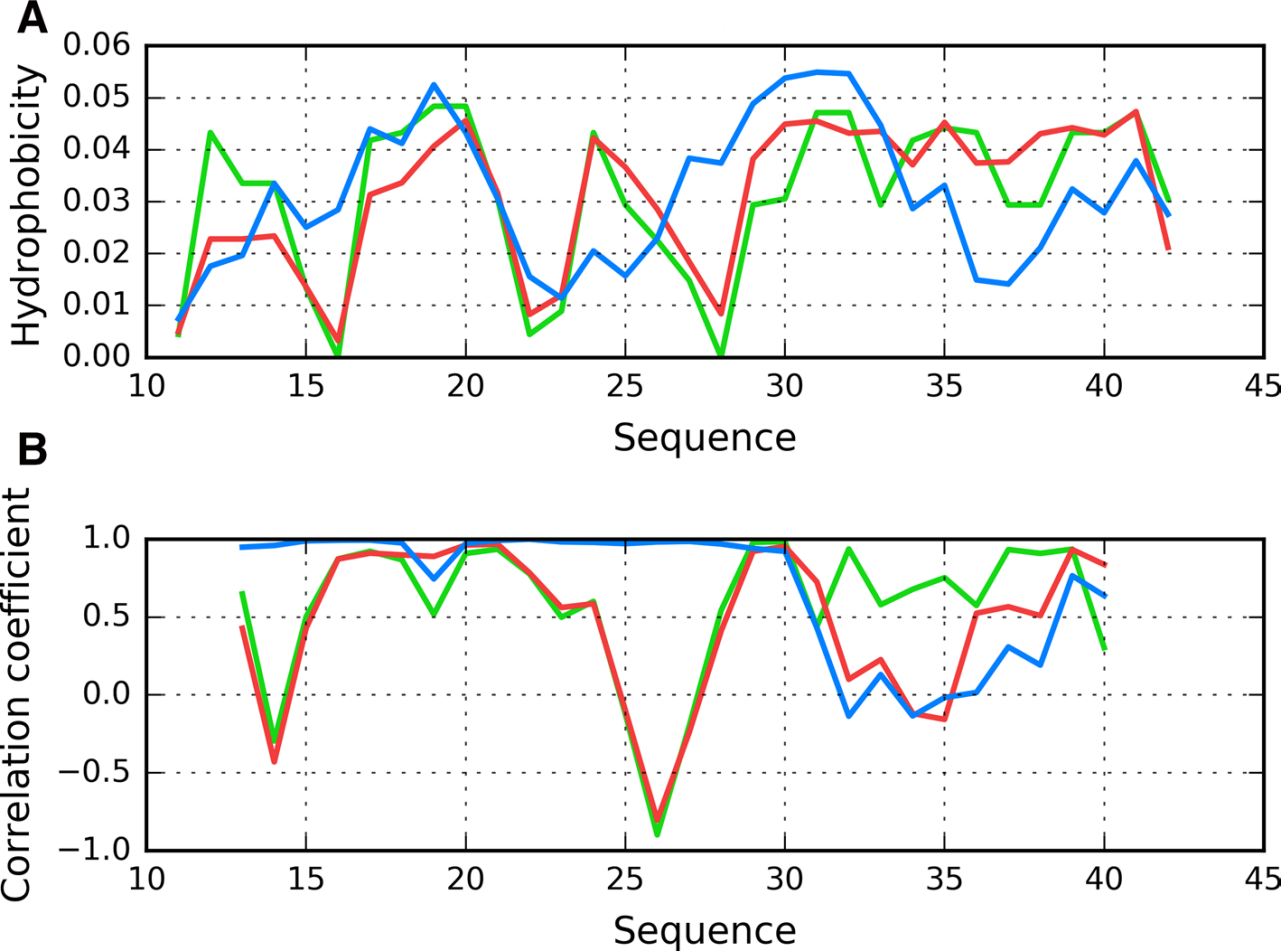
Chain F analyzed as a component of the fibril: **A** T (blue), O (red) and H (green) hydrophobicity distributions; **B** correlation coefficients (HvO: blue, HvT: red, TvO: green) calculated for a 5 aa moving frame (in overlapped system). The indicated position on X axis represents the central residue in a given frame (i. e. 20 corresponds to residue 20 in the 18–19–20–21–22 frame)

**Fig. 4:**
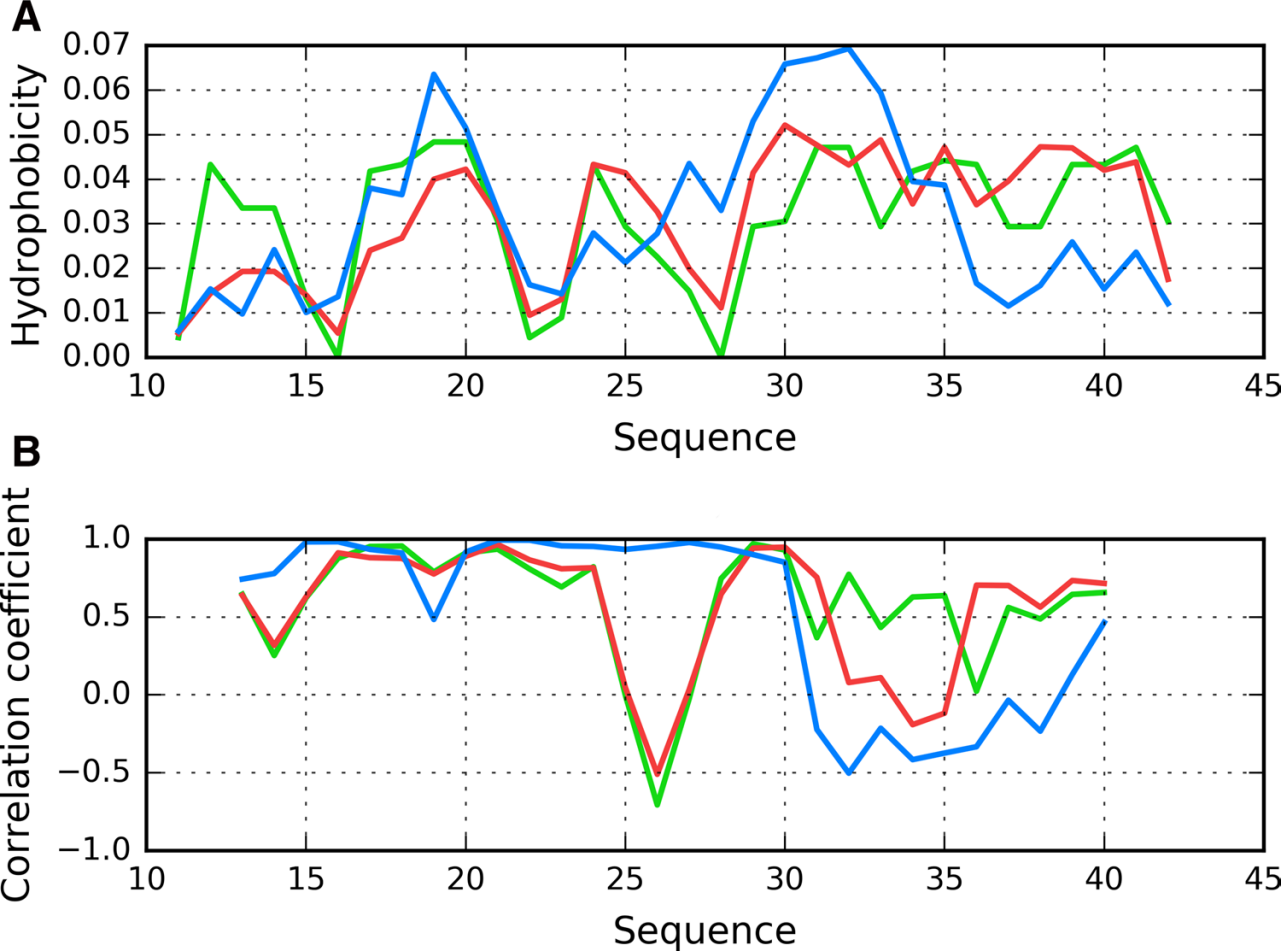
Chain F analyzed as an individual molecule: **A** T (blue), O (red) and H (green) hydrophobicity distributions; **B** correlation coefficients (HvO: blue, HvT: red, TvO: green) calculated for a 5 aa moving frame (in overlapped system). The indicated position on X axis represents the central residue in a given frame (i. e. 20 corresponds to residue 20 in the 18–19–20–21–22 frame)

Since our analysis involves a complex which consists of a finite number of peptides, it does not accurately reflect the theoretical capability for unrestricted propagation. For this reason, we have singled out chain F (the central one) as representative of the fibril’s structure. This chain was subsequently analyzed from two perspectives: as a part of the fibril (with 3D Gaussian fitted to the whole complex) and as an individual molecule (with 3D Gaussian fitted only to this chain and with disregard of other chains during O profile calculations). Figures 3a and 4a illustrate the theoretical, observed and intrinsic distributions for chain F. Besides sections where T is somewhat aligned with O (as well as with H), there are areas where both distributions diverge notably. In order to identify these discordant sections, we calculated HvT, TvO and HvO correlation coefficients for individual 5 aa fragments using a moving frame approach, as shown in Fig. 3b and in Fig. 4b. These charts reveal fragments where all three coefficients adopt relatively high values. These fragments (residues 5 through 11) may be regarded as accordant with the 3D Gaussian model—they evidence the sort of cooperation which is required throughout the whole protein for a monocentric hydrophobic core to emerge.

The remaining fragments exhibit discordance which, in extreme cases, may produce a conformation which is a polar opposite of theoretical predictions (negative values of TvO and HvT). In these cases, the observed distribution is driven by the “selfish” tendencies of each residue rather than by any tendency to produce a common hydrophobic core. Accordingly, we singled out fragments 11–16 and 24–28 as dominated by intrinsic hydrophobicity, while the 18–23 fragment may be described as locally accordant. Other fragments, while not exhibiting a clear preference for either T or H, are included in our analysis to enable comparisons with structures produced by in silico tools. Their variable status (from globular to amyloid-like) will be discussed on the basis of RD and correlation coefficients.

Profiles observed for both views of chain F similarly reveal the presence of unexpected local maximum in the localization of expected local minimum in fragment 22–28. The discordant position of Lys16 and particularly Lys28 destroy the expected maximum introducing local minimum. Taking into account that this characteristics is continued along the whole fibril. This is why the positions of lysines play the contradictory role in respect to centralization of hydrophobicity. Calculation for both forms reveals the unusual distribution which can be even treated as contradictory status in respect to usual characteristics observed in globular proteins.

### Comparative analysis of protein structures generated by in silico folding models

Structures generated by I-Tasser are labeled “I”, those generated by Robetta are labeled “R”, while those produced by the fuzzy oil drop software are labeled “F”. All structures are numbered, with their respective numbers affixed to the source label. Results are listed in order of increasing values of RD(T-O-R) for the whole molecule, meaning that F1 is the FOD output with lowest value, R1—Robetta’s, and so on.

Table 1 shows the full range of RD and correlation coefficients calculated for the entire molecule and for selected fragments. The list also includes chain F seen as a part of the fibril (labeled Af—amyloid/fibril, to avoid confusion with FOD results) and as an individual molecule (Ai—amyloid/individual). Table 1 also shows the status of the 29–42 fragment, even though—while somewhat disordered—it does not resemble an amyloid seed [12].

**Table 1:** Hydrophobicity-based parameters characterizing the structure of chain F from 2MXU in following forms: Af—when analyzed as a part of the fibril, Ai—when analyzed as an individual molecule, I#—as a result of simulation with I-Tasser, R#—as a result of simulation with Robetta, F#—as a result of simulation with FOD model

FORM	RD 11–42		CC 11–42			RD 11–16		CC 11–16			RD 17–23	
	T-O-R	T-O-H	HvT	TvO	HvO	T-O-R	T-O-H	HvT	TvO	HvO	T-O-R	T-O-H
F1	0.234	0.175	0.440	0.850	0.531	0.335	0.059	0.188	0.653	0.807	0.138	0.119
F2	0.240	0.181	0.252	0.866	0.370	0.184	0.081	0.309	0.961	0.486	0.194	0.155
F3	0.242	0.156	0.139	0.854	0.415	0.249	0.050	0.380	0.835	0.773	0.169	0.083
R1	0.256	0.260	0.589	0.776	0.626	0.275	0.115	0.807	0.828	0.977	0.330	0.326
R2	0.283	0.260	0.659	0.777	0.676	0.182	0.137	0.751	0.941	0.810	0.354	0.329
R3	0.320	0.280	0.447	0.746	0.716	0.417	0.123	0.376	0.485	0.953	0.387	0.384
I1	0.376	0.240	0.258	0.696	0.687	0.346	0.035	− 0.208	0.717	0.257	0.304	0.248
I2	0.428	0.287	0.272	0.612	0.709	0.757	0.172	− 0.512	− 0.111	− 0.597	0.473	0.348
**R4**	0.457	0.371	0.166	0.477	0.651	0.385	0.167	0.226	0.628	0.871	0.187	0.191
I3	0.487	0.341	0.167	0.468	0.640	0.707	0.189	− 0.481	0.015	0.531	0.757	0.509
**Ai**	0.536	0.519	0.408	0.567	0.698	0.488	0.220	0.494	0.500	0.831	0.121	0.230
F4	0.555	0.285	− 0.073	0.412	0.329	0.534	0.064	− 0.533	0.566	0.084	0.447	0.197
F5	0.559	0.226	0.154	0.391	0.260	0.533	0.054	− 0.005	0.190	0.606	0.874	0.275
**R5**	0.660	0.521	0.172	0.254	0.641	*0.655*	*0.201*	− *0.474*	− *0.075*	*0.739*	0.675	0.458
**Af**	0.680	0.756	0.246	0.363	0.821	0.506	0.562	0.121	0.257	0.966	0.133	0.473
**I4**	0.715	0.392	− 0.262	0.415	0.311	*0.903*	*0.176*	− *0.469*	− *0.452*	*0.788*	0.464	0.166
**I5**	0.768	0.610	− 0.138	0.014	0.558	0.822	0.170	0.259	− 0.118	0.680	*0.826*	*0.720*

FORM	CC 17–23			RD 24–28		CC 24–28			RD 29–42		CC 29–42		
	HvT	TvO	HvO	T-O-R	T-O-H	HvT	TvO	HvO	T-O-R	T-O-H	HvT	TvO	HvO
F1	0.632	0.939	0.716	0.269	0.251	0.845	0.866	0.965	0.264	0.189	− 0.030	0.824	− 0.307
F2	0.489	0.897	0.579	0.429	0.251	− 0.159	0.562	0.559	0.260	0.174	− 0.243	0.880	− 0.526
F3	0.092	0.908	0.440	0.353	0.179	0.521	0.717	0.912	0.168	0.123	− 0.386	0.910	− 0.306
R1	0.706	0.731	0.739	0.116	0.074	0.910	0.974	0.972	0.537	0.360	− 0.248	0.600	− 0.057
R2	0.608	0.653	0.863	0.367	0.091	0.566	0.718	0.937	0.433	0.431	0.675	0.650	0.373
R3	0.303	0.637	0.846	0.129	0.057	0.906	0.957	0.978	0.322	0.264	0.213	0.872	0.136
I1	0.416	0.808	0.826	0.530	0.092	− 0.167	0.243	0.854	0.618	0.675	0.259	0.674	0.514
I2	0.284	0.433	0.741	0.217	0.047	0.525	0.828	0.867	0.622	0.648	0.313	0.688	0.506
**R4**	0.766	0.959	0.632	*0.705*	*0.192*	− *0.641*	− *0.633*	*0.978*	0.575	0.486	− 0.199	0.536	0.085
I3	− 0.282	− 0.314	0.847	0.336	0.121	0.187	0.829	0.526	0.576	0.528	0.544	0.703	0.317
**Ai**	0.907	0.925	0.888	*0.669*	*0.382*	− *0.509*	− *0.706*	*0.954*	0.864	0.789	0.100	0.504	0.110
F4	0.388	0.453	0.288	0.435	0.237	− 0.499	0.464	0.435	0.711	0.582	− 0.062	0.433	− 0.260
F5	0.155	− 0.762	− 0.047	0.606	0.135	− 0.633	0.019	0.628	0.536	0.322	− 0.337	0.566	− 0.739
**R5**	0.065	− 0.089	0.665	0.226	0.037	0.726	0.872	0.952	0.810	0.784	0.266	0.410	0.322
**Af**	0.969	0.913	0.968	*0.731*	*0.557*	− *0.803*	− *0.898*	*0.982*	0.845	0.776	0.141	0.384	0.404
**I4**	− 0.879	0.444	− 0.194	*0.702*	*0.133*	− *0.787*	− *0.840*	0.949	0.765	0.660	− 0.045	0.705	− 0.104
**I5**	− *0.750*	− *0.454*	0.662	0.330	0.045	0.175	0.781	0.719	0.871	0.800	0.013	− 0.022	− 0.093

Sequential numbers (1–5) after I, R and F sort models obtained with given program in order of increasing value of RD(T-O-R) calculated for the whole chain (residues 11–42). Parameters are given as as follows: RD for T-O-R and T-O-H variants, correlation coefficients (CC) for relations H versus T (HvT), T versus O (TvO) and H versus O (HvO). Id of structures given in bold distinguish fragment classified as amyloid; values italic are examples of forms close to amyloid form, understood as those expressing negative CC value of HvT and/or TvO with high CC value of HvO and with high RD values (in both variants)

**Fig. 5:**
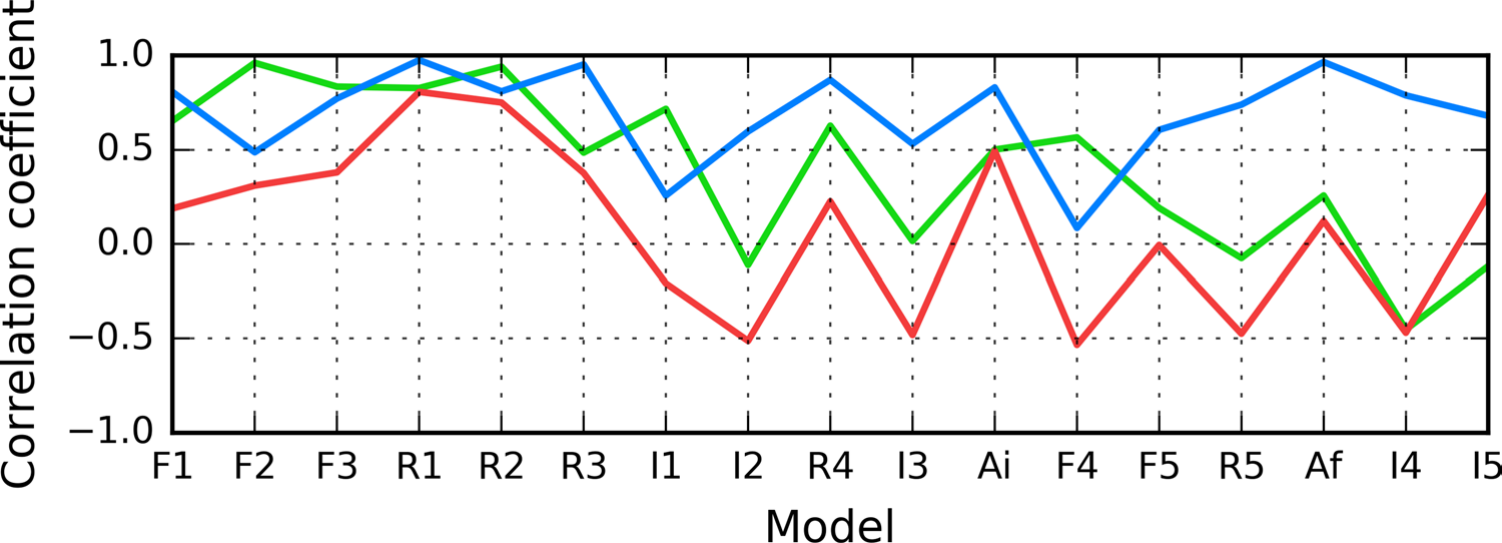
HvO (blue), HvT (red), TvO (green) calculated for residues 11–16 in successive structures as listed in Table 1

**Fig. 6:**
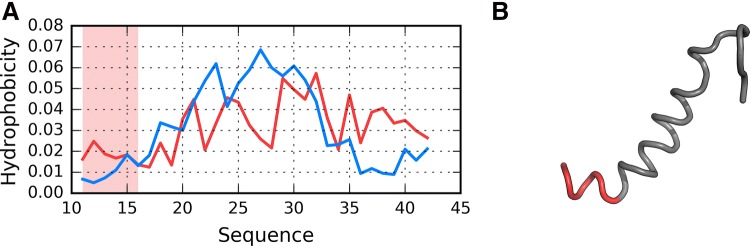
Presentation of structure I4: **A** theoretical (T: blue) and observed (O: red) hydrophobicity profiles; **B** 3D view. Red highlight in **A** and red fragment in **B** correspond to 11–16 residue range

According to Fig. 5, in models F1–R2, status of the 11–16 fragment is consistent with the 3D Gaussian distribution. This property, typical for globular proteins, fails to hold for model I1. In contrast, models R5 and I5 exhibit clear amyloid-like characteristics. Their HvO coefficients reach very high values, while the remaining coefficients are negative, indicating strong influence of intrinsic hydrophobicity. The set includes chain F analyzed as part of the complex (Af). Notably, under FOD classification criteria, model I4 exhibits the strongest amyloid affinity of the 11–16 fragment from among all analyzed structures. Its hydrophobicity profiles and 3D representation are shown on Fig. 6.

**Fig. 7:**
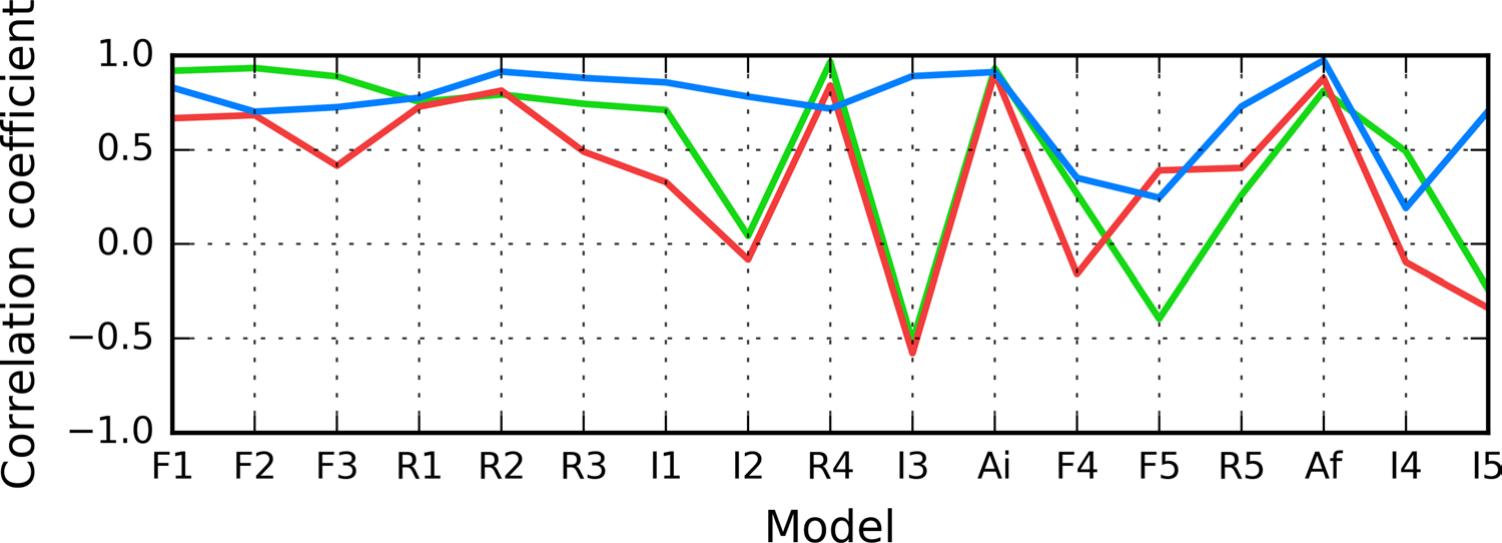
HvO (blue), HvT (red), TvO (green) calculated for residues 16–22 in successive structures as listed in Table 1

The summary shown in Fig. 7 shows that the 16–22 fragment in structure I3 exhibits amyloid-like characteristics, while structures F1–R4 are generally consistent with the theoretical distribution.

**Fig. 8:**
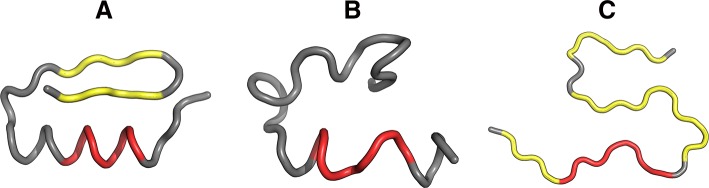
3D view of structures R3 (**A**), I3 (**B**) and chain F (**C**) with 16–22 fragment colored red. Yellow fragments mark the locations of beta sheets (partially covered with red in **C**)

Structures shown in Fig. 8 visualize the hypothesis that the folding following intrinsical hydrophobicity directs the process toward the amlyoid-like structural forms. Two structural forms compared in Fig. 8 show that the 16–22 fragment may adopt a helical conformation, yet in an amyloid it becomes beta-like. In addition to that, these two models prove that the Aβ(11–42) sequence may, in fact, produce a globule which can be seen to break apart as it transformation into an uncoiled loop (Fig. 8b) on its way to the amyloid form.

**Fig. 9:**
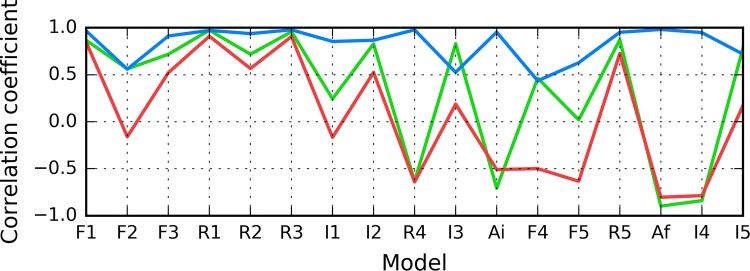
HvO (blue), HvT (red), TvO (green) calculated for residues 24–28 in successive structures as listed in Table 1

**Fig. 10:**
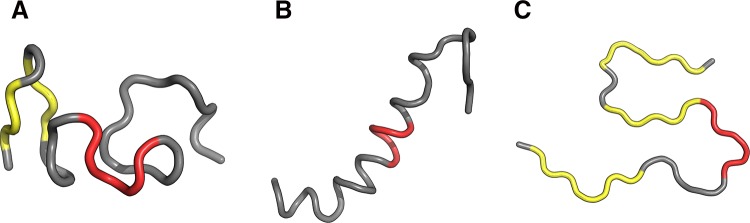
3D view of structures R4 (**A**), I4 (**B**) and chain F (**C**) with 24–28 fragment colored red. Yellow fragments mark the locations of beta sheets

As illustrated in Fig. 9, the fragment at 24–28 becomes amyloid-like in structures Af (chain F in fibril) and I4. Analysis of distribution charts in this figure shows that in models F1, R1, R2 and R3 the fragment retains globular characteristics, while in model R4 it also resembles an amyloid. Sample 3D structures are visualized in Fig. 10.

**Fig. 11:**
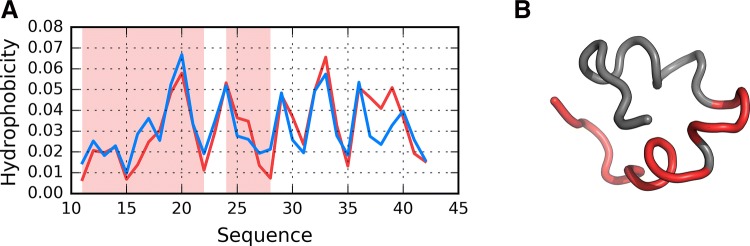
Presentation of structure F1: **A** theoretical (T: blue) and observed (O: red) hydrophobicity profiles; **B** 3D view. Red highlights in **A** and red fragments in **B** correspond to 11–16, 16–22 and 24–28 residue ranges

**Fig. 12:**
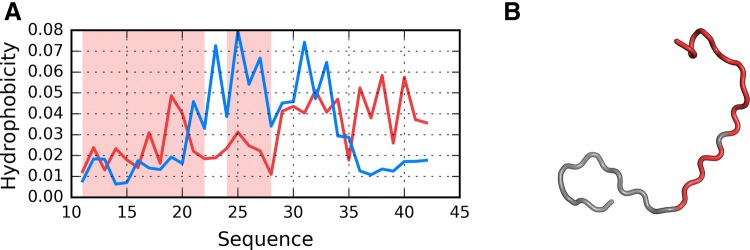
Presentation of structure I5: **A** theoretical (T: blue) and observed (O: red) hydrophobicity profiles; **B** 3D view. Red highlights in **A** and red fragments in **B** correspond to 11–16, 16–22 and 24–28 residue ranges

**Fig. 13:**
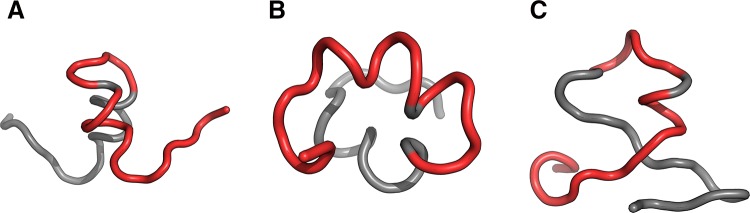
3D presentation of selected structures exhibiting RD(T-O-R) values calculated for the whole chain below 0.4 (exhibiting good accordance with FOD model): F2 (**A**), F3 (**B**) and I1 (**C**) with fragments 11–16, 16–22 and 24–28 in red

**Fig. 14:**
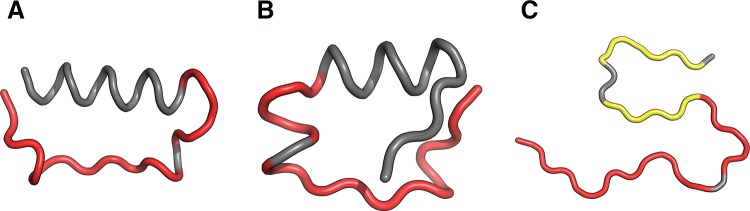
3D presentation of structures accordant with the FOD model: R1 (**A**), R2 (**B**) contrasted with the amyloid structure–chain F (**C**)—with fragments 11–16, 16–22 and 24–28 in red. Yellow fragments mark the locations of beta sheets

From among the analyzed structures, F1’s chain exhibits a particularly low RD(T-O-R) value, as shown in Fig. 11, where globular form was received with very little discordance between T and O profiles. In contrast, the highest RD(T-O-R) value was recorded for model I5’s chain, with its T and O profiles illustrated in Fig. 12. To allow further analysis and comparison with other models, a selection of them is shown in Figs. 13 and 14. They visualize what kind of wide variety of structures can be produced by highly specialized programs.

In summary, we can single out structure I5 as potentially susceptible to linear propagation via complexation of identically folded polypeptides. Structure I5 (random coil structural form) also exhibits a propensity for complexation, however its distribution of hydrophobicity does not reveal the characteristic sinusoidal pattern observed in amyloids [12].

One can conclude that the main candidate for amyloid transformation in the Aβ(11–42) are two fragments: 11–16 and 24–28. It can be seen in Table 1: the underlined sets of parameters denote models close to amyloid form: I4 and R4. These positions do not satisfy the condition of high value of RD(T-O-H). High value of this parameter requires a multi-chain complex as it is rather unusual for an isolated chain. This conclusion is correct on the condition of fuzzy oil drop acceptance as the method to trace the hydrophobic core identification and its transformation. This is why conclusion can be limited just to such a case.

## Discussion

Summing up the presented results, we can state that the Aβ(11–42) polypeptide may theoretically adopt various structural forms, including tightly packed globules characterized by high solubility (good agreement between O and T distributions). The set of candidate structures produced by I-Tasser and Robetta is highly diverse—from coherent globules all the way to disordered folds. Of particular note are the structured generated using the FOD model, where the presence of an external force field (aqueous solvent) drives the folding process towards the generation of a monocentric hydrophobic core—even though, under certain (so far unknown) conditions, this model may also produce strongly discordant structures.

Some of the obtained structures, e.g. I5 with RD(T-O-R) = 0.768, appears capable of forming complexes with other identically folded chains. The structure in question includes fragments which may be regarded as seeds of a conformational pattern guided by the intrinsic hydrophobicity of individual residues (negative correlation coefficients—see Table 1).

The interpretation provided in [33, 34] suggests that external conditions may support the misfolding not supporting the formation of centric hydrophobic core. The form of all hydrophobicity profiles as observed in amyloid forms analyzed here mark the positions of lysines as highly discordant in respect to what is expected by T distribution. The position of lysines introduces sharp local minimum in O in location where the local maximum is expected (due to the lowest intrinsic hydrophobicity in the scale). Especially position Lys28 introduces significant discordance. In this context, it is surprising to note the emergence of hydrophilic bands formed by linearly arranged lysine residues, as such conformations should be deterred through optimization of electrostatic interactions—and yet they can be observed under experimental conditions. In fragments characterized by negative correlation coefficients the central position is frequently occupied by lysine. When multiple fragments aggregate in a linear manner, these lysines come into close contact with one another, forming a hydrophilic band. This shows that hydrophobic forces override electrostatic interactions, and that disruptions in the external force field may produce a conformation which depends on the intrinsic hydrophobicity of participating residues. The above interpretation provides indirect confirmation of the correctness of the fuzzy oil drop model, which, under ordinary circumstances, leads to the emergence of a monocentric hydrophobic core encapsulated by a hydrophilic “shell”. The importance of hydrophobic interactions for seeding amyloid transformation has already been noted in [35, 36]. In [36] the authors suggest that amyloid transformation may result from insufficient influence of the external force field (water) upon the folding process. This conclusion is consistent with the interpretation of results obtained using the fuzzy oil drop model, as presented in this paper.

In conclusion based on the presented model the weakening of standard external force field (water environment) prevents it from driving the forlding process toward centralization of hydrophobic residues and allows the intrinsic hydrophobicity to dominate.

The effect in this case is the micellarization. In particular, a ribbon-like micelle is preferred despite non-favorable interaction of charged residues arranged in electrostatically suboptimal form. The arguments for this conclusion are coming from in vitro experiments—especially shaking, which introduces air to water and—in consequence—much higher presence of inter-phase-related order of water molecules. This is why the structure of water in its standard form as well as under influence of external factors (not necessarily of chemical character) should be in focus of research oriented on amyloid transformation.
